# The Herbal Medicine *Scutellaria-Coptis* Alleviates Intestinal Mucosal Barrier Damage in Diabetic Rats by Inhibiting Inflammation and Modulating the Gut Microbiota

**DOI:** 10.1155/2020/4568629

**Published:** 2020-11-08

**Authors:** Boxun Zhang, Rensong Yue, Yuan Chen, Xiaoying Huang, Maoyi Yang, Jiacheng Shui, Yuliang Peng

**Affiliations:** ^1^Hospital of Chengdu University of Traditional Chinese Medicine, Chengdu 610075, China; ^2^Shaanxi University of Chinese Medicine, Xianyang 712046, China

## Abstract

Recent studies have confirmed that increased intestinal permeability and gut-origin lipopolysaccharide (LPS) translocation are important causes of metabolic inflammation in type 2 diabetes (T2D), but there are no recognized therapies for targeting this pathological state. *Scutellaria baicalensis* and *Coptis chinensis* are a classic herbal pair often used to treat diabetes and various intestinal diseases, and repair of intestinal barrier damage may be at the core of their therapeutic mechanism. This study investigated the effects of oral administration of *Scutellaria-Coptis* (SC) on the intestinal mucosal barrier in diabetic rats and explored the underlying mechanism from the perspective of anti-inflammatory and gut microbiota-modulatory effects. The main results showed that, in addition to regulating glycolipid metabolism disorders and inhibiting serum inflammatory factors, SC could also upregulate the expression levels of the tight junction proteins claudin-1, occludin, and zonula occludens (ZO-1), significantly improve intestinal epithelial damage, and inhibit excessive LPS translocation into the blood circulation. Furthermore, it was found that SC could reduce the levels of the inflammatory factors interleukin-1*β* (IL-1*β*), IL-6, and tumour necrosis factor-*α* (TNF-*α*) in intestinal tissue and that the anti-inflammatory effects involved the TLR-4/TRIF and TNFR-1/NF-*κ*B signalling pathways. Moreover, SC had a strong inhibitory effect on some potential enteropathogenic bacteria and LPS-producing bacteria, such as Proteobacteria, Enterobacteriaceae, *Enterobacter*, *Escherichia-Shigella*, and *Enterococcus*, and could also promote the proliferation of butyrate-producing bacteria, such as Lachnospiraceae and Prevotellaceae. Taken together, the hypoglycaemic effects of SC were related to the protection of the intestinal mucosal barrier, and the mechanisms might be related to the inhibition of intestinal inflammation and the regulation of the gut microbiota.

## 1. Introduction

Globally, type 2 diabetes (T2D) has become a major threat to human health. According to the diabetes map released by the International Diabetes Federation (IDF), there are 463 million patients with diabetes worldwide among people aged 20–79 years, with T2D accounting for the vast majority of diabetes cases (approximately 90%) [1]. In recent years, accumulating evidence has indicated that chronic inflammation plays a significant role in the occurrence and development of T2D, and anti-inflammatory administration is a potential approach to treatment [2–4]. Clinical studies found that proinflammatory factors such as interleukin-6 (IL-6) and C-reactive protein (CRP) were significantly higher for the population who developed diagnosed diabetes over the follow-up period, and to some degree, these indicators could be used as powerful predictors of T2D [5, 6]. Experimental studies have confirmed that inflammatory cytokines could inhibit the phosphorylation of insulin receptor substrate proteins and promote the M1-like polarity shift of islet macrophages, causing insulin resistance (IR) and islet dysfunction [7, 8].

The production of inflammation in T2D is closely related to the “leakage” of intestinal lipopolysaccharide (LPS) [9]. LPS is a vital component of the outer membrane of intestinal gram-negative (G−) bacteria, and when intestinal permeability increases, excessive LPS can enter the blood circulation and trigger the inflammatory cascade reaction [10]. The intestinal epithelial mucosa is a selective permeable barrier mainly composed of closely arranged intestinal epithelial cells and tight junction complexes between cells [11]. Several clinical studies have found that intestinal mucosa damage is a common pathological state in patients with T2D, and it tends to be more serious in patients with a long disease course and poor blood glucose control [12–15]. Recent studies have shown that some intestinal microecological agents could improve metabolic inflammation by protecting the intestinal barrier function and inhibiting LPS translocation; therefore, targeting the intestinal barrier is a promising strategy for treating T2D [16].

The gut microbiota is a key factor in maintaining the integrity of the intestinal epithelium. A series of studies confirmed that some beneficial bacteria belonging to *Bifidobacterium*, *Akkermansia*, and *Lactobacillus* could play roles in intestinal mucosa protection and the decrease in their abundance is related to the occurrence of various intestinal diseases and systemic low-grade inflammation [16–18]. Butyrate, the fermentation product of dietary fibre, can provide energy for intestinal epithelial cells [19], and some evidence has suggested that butyrate can not only directly increase the intestinal expression of mucin 2 (MUC2) and tight junction proteins but can also play an anti-inflammatory role by inhibiting the expression of the transcription factor nuclear factor-kappa B (NF-*κ*B) [19]. In contrast, some potential pathogens, such as adhesive-invasive *Escherichia coli,* might trigger an inflammatory response and disturb intestinal mucosa homeostasis [20]. A metagenome-wide association study confirmed that the gut microbiota of patients with T2D was characterized by decreased butyrate-producing bacteria and increased opportunistic pathogens [21], thereby tending to result in intestinal mechanical barrier lesions and allowing the translocation of bacteria or LPS through the intestinal mucosa.

Except for some intestinal bacteria, the production of excessive LPS in the gut can also activate an inflammatory reaction and destroy the homeostasis of intestinal barrier function [22]. The combination of LPS and its receptor toll-like receptor-4 (TLR-4) can activate the downstream myeloid differentiation factor 88 (MyD88) or TIR-domain-containing adapter-inducing interferon-*β* (TRIF) signalling pathways and then promote the translocation of NF-*κ*B and the production of various inflammatory factors [23]. In addition, the enrichment of proinflammatory cytokines can further aggravate intestinal mucosa damage. For example, tumour necrosis factor-*α* (TNF-*α*) can activate the inflammatory response and induce cell apoptosis via a receptor-dependent pathway [24] and increase intestinal permeability by altering the structure and function of tight junction proteins [25].

Nowadays, more and more attention has been paid to the antidiabetic effects of herbal medicines and their extracts. For example, in Thailand and some other Asian countries, *Rhinacanthus nasutus* (L.) Kurz has traditionally been used in treating various disorders including diabetes, and rhinacanthins, the extract of *R. nasutus*, had been confirmed to have significant anti-hyperglycemia, anti-hyperlipidemia, and antiobesity effects, which was considered as a potential candidate for antidiabetic drug development [26–28]. In China, the *Scutellaria*-*Coptis* (SC) herbal pair is the basic component of many herbal formulas used for treating diabetes [29]. Pharmacological studies have confirmed that the flavonoids in *Scutellaria baicalensis* and the alkaloids in *Coptis chinensis* are the main material basis for ameliorating T2D, and their therapeutic mechanisms involve anti-inflammatory effects and the regulation of the gut microbiota [30, 31]. In addition, some studies have shown that SC or its active ingredients could also protect the intestinal barrier function in both intestinal and systematic diseases [32–34]. Therefore, we conducted this study to determine whether SC could improve the metabolic inflammation of T2D rats by repairing the intestinal barrier and preventing the leakage of intestinal LPS, and we explored the underlying mechanism from the perspective of anti-inflammatory and gut microbiota-regulatory effects.

## 2. Materials and Methods

### 2.1. Animals, Modelling, and Groups

Male Sprague Dawley (SD) rats weighing 180–200 g were purchased from Dossy Experimental Animal Co., Ltd. (Chengdu, China) and were raised in SPF experimental conditions in the animal facility of the Clinical Medicine College of Chengdu University of T.C.M. The rats were kept in a humanized environment of 20 ± 2°C, humidity 60 ± 5%, and a 12-h light/dark cycle, with *ad libitum* access to normal chow diet and purified water. After acclimatization for 2 weeks, the rats were randomly divided into a normal control group (NC group) and a high-fat group (HF group) and were fed a normal chow or high-fat diet (Table S1). Eight weeks later, the rats received an injection of streptozotocin (STZ, Sigma Chemical Corp, USA) at a dose of 30 mg/kg for the HF group and an equal dose of NaCl 0.9% for the NC group. After 72 h, serum glucose in the tail vein was determined using a Johnson and Johnson blood glucose meter and test strips (Johnson and Johnson, USA), and rats with blood glucose values of 16.7–30 mmol/L were selected as the T2DM animal model [35, 36]. Diabetic rats were further randomly divided into 5 groups (*n* = 7 per group): a diabetes control group (DC group), a diabetes with high-dose *Scutellaria-Coptis* group (DHSC group), a diabetes with middle-dose *Scutellaria-Coptis* group (DMSC group), a diabetes with low-dose *Scutellaria-Coptis* group (DLSC group), and a diabetes with metformin group (DME group). This animal study was approved by the experimental animal ethics committee of Chengdu University of T. C. M.

### 2.2. Preparation of SC and Treatment Methods

*Scutellaria baicalensis* and *Coptis chinensis*-dispensing granules were purchased from Sichuan Neo-Green Pharmaceutical Technology Development Co., Ltd. (Chengdu, China), and the main bioactive compounds of SC were identified and determined by high-performance liquid chromatography (Figure S1). The high, medium, and low dosages in the SC treatment groups were 8.4, 4.2, and 2.1 g kg^−1^ day^−1^, respectively. Metformin, the first-line medication for T2DM, was chosen as the positive control, and the dose was 178.5 mg/kg. Rats in the NC and DC groups were given distilled water by gavage. The dose of gavage was adjusted weekly according to the weight of the rats, and the intervention time was 12 weeks.

### 2.3. Oral Glucose Tolerance Test and Insulin Tolerance Test

At 12 weeks, the rats were fasted for 12 h (overnight) followed by the performance of an oral glucose tolerance test (OGTT). After measuring fasting blood glucose (FBG), rats were intragastrically administered 2 g/kg glucose solution, and blood glucose was measured at 30, 60, 90, and 120 minutes. Three days later, the insulin tolerance test (ITT) was performed by injecting 0.75 U/kg human insulin (Novolin, Novo Nordisk (China) Pharmaceutical Co., Ltd. Tianjin, China) after a 6-h fast, and blood glucose was measured at 0, 30, 60, 90, and 120 minutes after intraperitoneal administration. Blood glucose was determined from tail blood using a glucometer (Ultra Vue, Johnson and Johnson (China) Co., Ltd., Shanghai, China), and the results were evaluated by calculating the area under the curve (AUC).

### 2.4. Biological Sample Collection and Preparation

At the end of 12 weeks of treatment, faecal samples were obtained by stimulating the anus of rats and were then stored in sterile cryopreservation tubes. After a 12-h fast, anaesthesia was conducted by intraperitoneal injection with 50 mg/kg pentobarbital sodium, and samples of femoral artery blood, ileum, and colon tissues were collected under sterile conditions. The blood sample was kept for two hours, centrifuged at 3000 r/min for 15 minutes, and refrigerated at −20°C. The tissue sample was divided into two parts and stored in 4% formaldehyde solution or in a −80°C freezer.

### 2.5. Biochemical Assays and ELISA Test

Serum total cholesterol (TC), total triglycerides (TG), low-density lipoprotein cholesterol (LDL-C), and high-density lipoprotein cholesterol (HDL-C) were detected by biochemistry reagent kits (Nanjing Jiancheng Bio-Engineering Institute Co., Ltd. Nanjing, China) and an automated biochemical analyser (BS-600, Shenzhen Mindray Biomedical Electronic Co., Ltd. Shenzhen, China). Serum-free fatty acids (FFAs), insulin (INS), diamine oxidase (DAO), D-lactate (D-LA), LPS, and interleukin-1*β* (IL-1*β*), IL-6, and TNF-*α* were assessed using corresponding ELISA kits (Shanghai Zhuocai Biotechnology Co., Ltd. Shanghai, China) and a multifunctional enzyme labelling tester (Epoch, American Berten Instruments Co., Ltd. Vermont, America). The specific experimental process was strictly in accordance with the manufacturer's protocols. To further evaluate the insulin resistance of rats in each group, we calculated the homeostasis model assessment-insulin resistance (HOMA-IR) index using the following formula [37]:

(1) $$\begin{array}{c} \text{HOMA}-\text{IR}=\frac{22.5}{\left(\text{FBG}\times \text{INS}\right)}. \end{array}$$

### 2.6. Histological Staining

Ileum and colon tissues were divided into 3 *μ*m sections through paraformaldehyde and paraffin embedding. Next, these samples were stained using haematoxylin and eosin (H&E) to assess the histological morphology. The main procedures included dehydration, pruning, embedding, sectioning, staining, and sealing. Tissue sections were observed and collected by a digital three-eye camera system (BA400 Digital, MOTIC China Group Co., Ltd. Xiamen, China).

### 2.7. Immunohistochemistry Analysis

The ileum tissues were fixed with 4% paraformaldehyde, embedded in paraffin, and sectioned. Rabbit polyclonal zonula occludens (ZO-1) antibody, rabbit polyclonal claudin-1 antibody, and rabbit polyclonal occludin antibody (Beijing Bioss Biotechnology Co., Ltd, Beijing, China) were used as the primary antibodies. The experimental process of immunohistochemistry mainly included dewaxing and rehydration, antigen repair, immune response, chemical staining, and dehydration sealing. Finally, the average optical density of the samples was measured using Image-Pro Plus image analysis software (Version 7.0, Media Cybernetics, USA). The positive cells are indicated by brown-yellow or yellow cytoplasm, and the negative cells are indicated by a blue nucleus.

### 2.8. Western Blot Assay

The key proteins of the TLR-4/TRIF and tumour necrosis factor receptor 1 (TNFR-1)/NF-*κ*B signalling pathways, including TLR-4, TRIF-related adaptor molecule (TRAM), TRIF, TNFR-1, TNFR-associated factor-2 (TRAF-2), receptor-interacting serine/threonine protein kinase-1 (RIPK-1), and NF-*κ*B-p65, were detected by western blot assay. Specifically, 50 mg of crushed ileum tissue and 200 *µ*L of precooled RIPA (Multi Sciences Biotech, Co., Ltd, Hangzhou, China) were added to the centrifuge tubes and homogenized, and a BCA protein quantitation assay (Multi Sciences Biotech, Co., Ltd, Hangzhou, China) was used to detect the total protein content of the homogenate supernatant. Next, equal amounts of protein samples in each group were separated by 10% sodium dodecyl sulfate-polyacrylamide gel electrophoresis (SDS-PAGE) and transferred to polyvinylidene difluoride (PVDF) membranes. After blocking the PVDF membranes with 5% skimmed milk powder solution at room temperature for 1 h, membranes were incubated with primary antibodies at appropriate concentrations overnight at 4°C. In this study, antibodies against TLR-4 and actin were purchased from Multi Sciences Biotech Co., Ltd. (Hangzhou, China). Antibodies against TRAM, TRIF, TNFR-1, RIPK-1, TRAF-2, and NF-*κ*B-p65 were purchased from Proteintech Group Co., Ltd. (Wuhan, China). On the second day, PVDF membranes were incubated with HRP-conjugated goat anti-rabbit secondary antibodies for 1 hour at room temperature, and a chemiluminescence detection system and X-ray film were then used for visualization and exposure, respectively.

### 2.9. 16S rRNA Sequencing

16S rRNA sequencing of the gut microbiota mainly included the following steps. First, genomic DNA was extracted from and detected in faecal samples. Second, specific primers with barcodes were synthesized to amplify the V4 region of 16S rDNA. The primer sequences were as follows: 515F (5′-GTGYCAGCMGCCGCGGTAA-3′); 806R (5′-GGACTACHVGGGTWTCTAAT-3′). Third, the PCR products were collected and quantified, and the purified amplification products were then mixed according to the requirements of sequencing quantity. Fourth, a sequencing library was constructed and sequenced with the PE250 mode of the HiSeq 2500 platform (Illumina, Inc., USA).

### 2.10. Statistical Analysis

SPSS 26.0 software was used to analyse all the results of this study, and data are expressed as the mean ± SD. One-way ANOVA was used to analyse significant differences between groups, and a value of *p* < 0.05 was considered statistically significant. R language and corresponding packages were used to analyse the composition and diversity of the gut microbiota.

## 3. Results

### 3.1. SC Improved Glycolipid Metabolism in T2DM Rats

**Figure 1:**
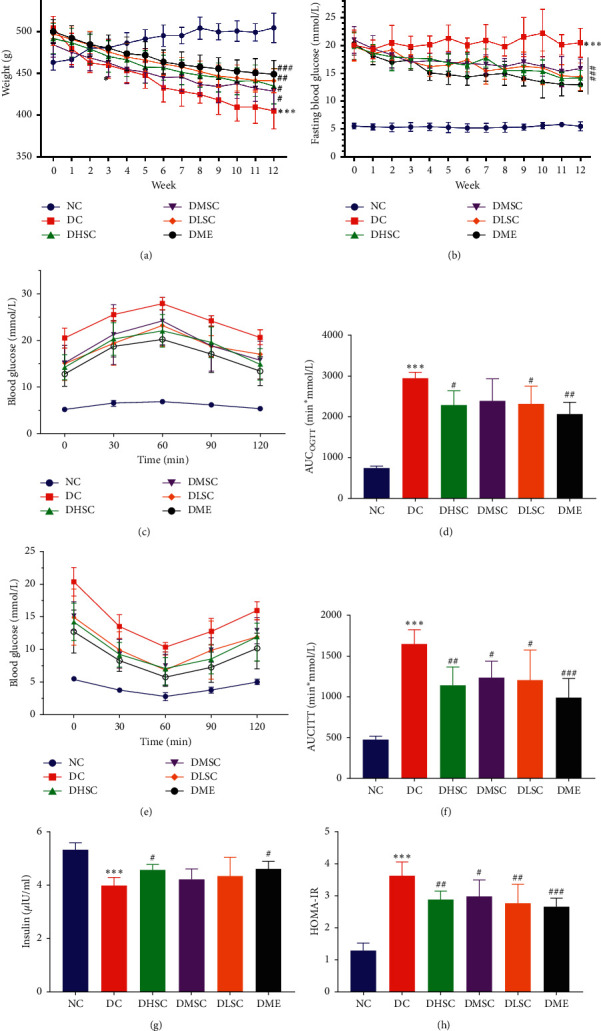
Effect of SC on body weight and glucose metabolism in T2D rats. (a) Body weight curve of rats in each group, (b) blood glucose curve of rats in each group, (c) the oral glucose tolerance test (OGTT), (d) the area under the curve (AUC) for the OGTT curves, (e) the insulin tolerance test (ITT), (f) the area under the curve (AUC) for the ITT curves, (g) the serum insulin level, and (h) the homeostasis ^*∗∗∗*^*p* < 0.001model assessment-insulin resistance (HOMA-IR) index. Data are expressed as the mean ± SD (*n* = 7 rats/group). vs. NC group, ^#^*p* < 0.05, ^##^*p* < 0.01, and ^###^*p* < 0.001 vs. DC group.

**Figure 2:**
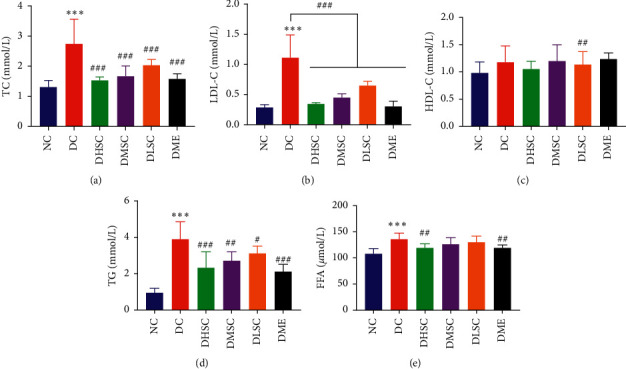
Effect of SC on lipid metabolism in T2D rats. (a) Serum total cholesterol (TC) level, (b) serum low-density lipoprotein cholesterol (LDL-C) level, (c) serum high-density lipoprotein cholesterol (HDL-C) level, (d) triglyceride (TG), and (e) serum free fatty acid (FFA) levels. Data are expressed as the mean ± SD (*n* = 7 rats/group). ^*∗∗∗*^*p* < 0.001 vs. NC group, ^#^*p* < 0.05, ^##^*p* < 0.01, and ^###^*p* < 0.001 vs. DC group.

In this study, the glycolipid metabolism of rats in each group was evaluated. Compared to the NC group, the body weight, FBG level, AUCs of the OGTT and ITT, index of homeostasis model assessment-insulin resistance (HOMA-IR) (Figures 1(a)–1(f) and 1(h)), and lipid metabolism indexes including total cholesterol (TC) (Figure 2(a)), low-density lipoprotein cholesterol (LDL-C) (Figure 2(b)), triglyceride (TG) (Figure 2(d)), and free fatty acids (FFAs) (Figure 2(e)) increased markedly, while the weight and serum INS levels (Figure 1(g)) showed a downward trend, and there was no significant difference in the serum high-density lipoprotein cholesterol (HDL-C) level (Figure 2(c)) among groups.

### 3.2. SC Inhibited the Serum Markers of Intestinal Mucosal Damage and Metabolic Inflammation in T2DM Rats

**Figure 3:**
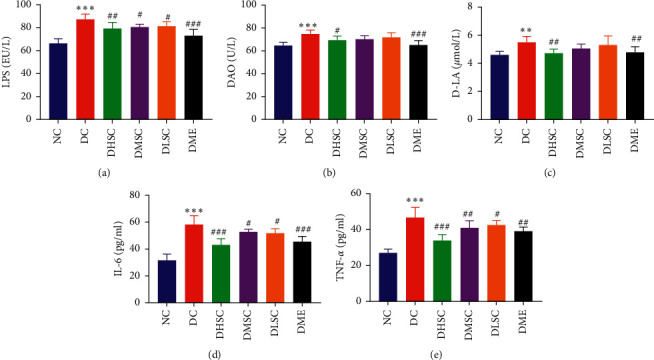
Effect of SC on serum markers of intestinal mucosal damage and metabolic inflammation in T2DM rats. (a) Serum lipopolysaccharide level, (b) serum diamine oxidase (DAO) level, (c) serum D-lactic acid (D-LA) level, (d) serum interleukin-6 (IL-6) level, and (e) serum tumour necrosis factor-*α* (TNF-*α*) level. Data are expressed as the mean ± SD (*n* = 7 rats/group). ^*∗∗*^*p* < 0.01, ^*∗∗∗*^*p* < 0.001 vs. NC group, ^#^*p* < 0.05, ^##^*p* < 0.01, and ^###^*p* < 0.001 vs. DC group.

Serum markers of intestinal mucosal damage, including LPS, DAO, and D-LA, and the serum inflammatory cytokines IL-6 and TNF-*α* were detected [31, 32]. Compared to the NC group, the data in the DC group increased significantly, indicating that there was no obvious intestinal barrier damage or LPS leakage in the T2DM rat model induced by a high-fat diet and STZ. However, the upward trend was reversed by SC and metformin treatment, and compared to medium- and low-dose SC, high-dose SC was more effective (Figures 3(a)–3(e)).

### 3.3. SC Repressed Intestinal Tissue Damage and Regulated the Expression of Tight Junction Proteins in T2DM Rats

**Figure 4:**
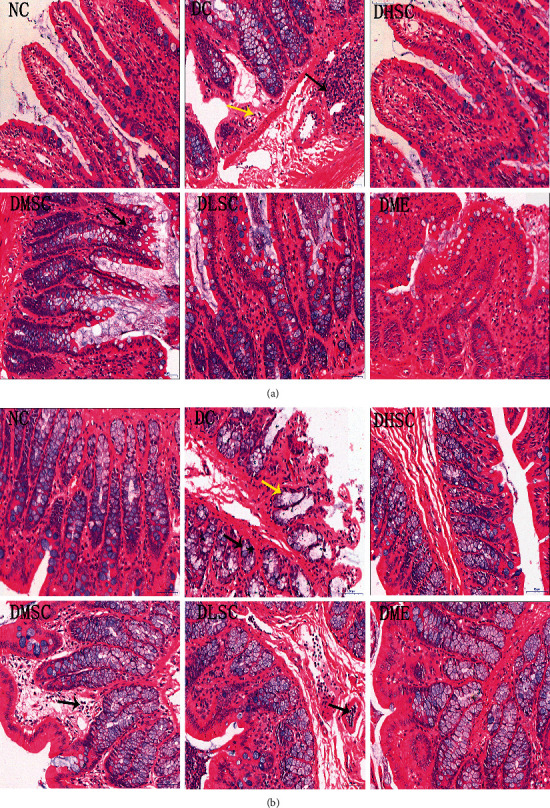
Effect of SC on the intestinal histopathology of T2DM rats. Representative HE-stained pictures of (a) ileum tissue and (b) colon tissue. *n* = 5 rats/group. The black arrow represents inflammatory cell infiltration, and the yellow arrow represents cell vacuolar degeneration.

To explore the intestinal pathological changes of T2DM rats induced by high-fat diet + STZ and the intestinal protective effects of SC, we observed HE staining pictures of the ileum (Figure 4(a)) and colon tissues (Figure 4(b)) via light microscopy. In the DC group, some exfoliated intestinal villi and chyme-like substances were mixed and accumulated in the intestinal cavity; some epithelial cells were degenerated and necrotic, accompanied by infiltration of inflammatory cells; in addition, atrophic intestinal glands and vacuolated cells could also be observed. After interventions with SC and metformin, these pathological features were improved.

**Figure 5:**
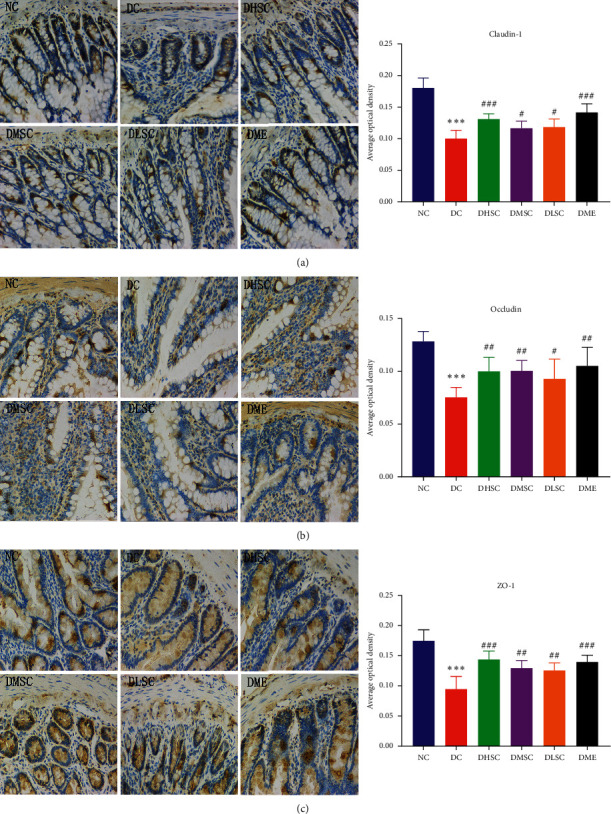
Effect of SC on intestinal tight junction proteins. Representative immunohistochemical image of (a) claudin-1 protein and the average optical density of positive staining, (b) occludin protein and the average optical density of positive staining, and (c) ZO-1 protein and the average optical density of positive staining. Data are expressed as the mean ± SD (*n* = 5 rats/group). ^*∗∗∗*^*p* < 0.001 vs. NC group, ^#^*p* < 0.05, ^##^*p* < 0.01, and ^###^*p* < 0.001 vs. DC group.

Claudin-1, occludin, and ZO-1 are representative intestinal tight junction proteins [38]. Immunohistochemistry findings revealed that, compared with the NC group, the protein expression levels in the DC group decreased significantly, and after treatment with SC and metformin, the protein expression showed an upward trend. In addition, the therapeutic effect of the high-dose group was better than that of the medium- and low-dose groups (Figures 5(a)–5(c)).

### 3.4. SC Inhibited the Expression of Intestinal Inflammatory Factors in T2DM Rats

**Figure 6:**
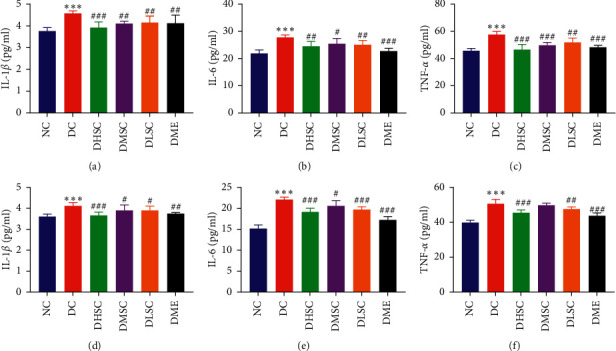
Effect of SC on the intestinal inflammatory response in diabetic rats. (a) Interleukin (IL)-1*β* level in the ileum, (b) IL-6 level in the ileum, (c) tumour necrosis factor-*α* (TNF-*α*) level in the ileum, (d) IL-1*β* level in the colon, (e) IL-6 level in the colon, and (f) TNF-*α* level in the colon. Data are expressed as the mean ± SD (*n* = 7 rats/group). ^*∗∗∗*^*p* < 0.001 vs. NC group, ^#^*p* < 0.05, ^##^*p* < 0.01, and ^###^*p* < 0.001 vs. DC group.

To explore the mechanism of SC in improving the intestinal mucosal barrier in T2DM rats, the inflammatory indexes of the ileum (Figures 6(a)–6(c)) and colon (Figures 6(d)–6(f)) were detected. The data showed that SC and metformin could effectively inhibit the expression of the intestinal inflammatory factors IL-1*β*, IL-6, and TNF-*α*. Compared with the middle- and low-dose groups, the SC high-dose group had a more significant effect, which showed that the anti-inflammatory effects of SC were dose-dependent. Comparing the ileum and colon data, we found that the expression of inflammatory factors in the ileum was higher in T2DM rats.

### 3.5. SC Inhibited Intestinal Inflammation through the TLR-4/TRIF and TNFR-1/NF-*κ*B Signalling Pathways

**Figure 7:**
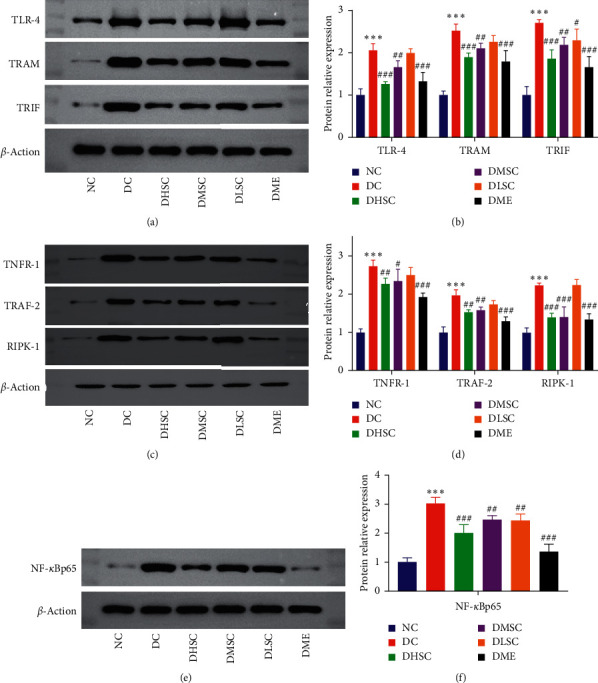
Effect of SC on key proteins in the TLR-4/TRIF and TNFR-1/NF-*κ*B signalling pathways. (a) Western blot (WB) images of the TLR-4, TRAM, and TRIF proteins and (b) relative protein expression of TLR-4, TRAM, and TRIF compared to *ß*-actin; (c) WB images of the TNFR-1, TRAF-2 and RIPK-1 proteins and (d) relative protein expression of TNFR-1, TRAF-2, and RIPK-1 compared to *ß*-actin; (e) WB images of the NF-*κ*B-p65 protein and (f) relative protein expression of NF-*κ*B-p65 compared to *ß*-actin. Data are expressed as the mean ± SD (*n* = 3 rats/group). ^*∗∗∗*^*p* < 0.001 vs. NC group, ^#^*p* < 0.05, ^##^*p* < 0.01, and ^###^*p* < 0.001 vs. DC group.

To further explore the molecular mechanism of SC in inhibiting inflammation of ileum tissue, the TLR-4/TRIF and TNFR-1/NF-*κ*B signalling pathway-related proteins TLR-4, TRAM, TRIF, TNFR-1, TRAF-2, RIPK-1, and NF-*κ*B-p65 were detected by western blotting (Figures 7(a)–7(f)). In the DC group, the expression levels of these proteins increased significantly, while in the treatment groups, especially the DHSC and DME groups, the expression levels of the above proteins were obviously inhibited, which suggested that the inhibition of intestinal inflammation by SC might be related to the TLR-4/TRIF and TNFR-1/NF-*κ*B signalling pathways.

### 3.6. Analysis of Richness and Diversity

**Figure 8:**
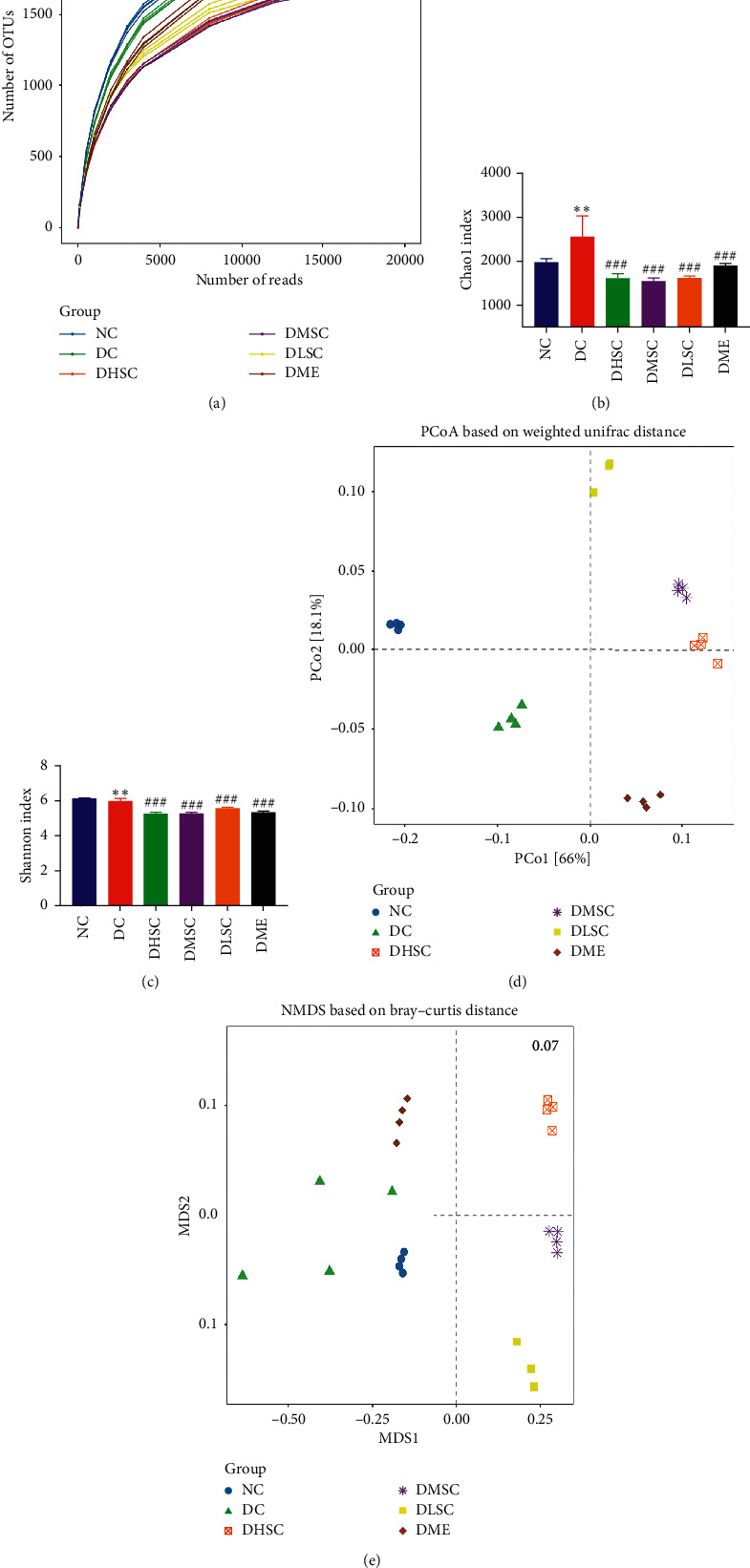
Effect of SC on the richness and diversity of the gut microbiota. (a) Rarefaction curves determined at the 97% similarity level, (b) the Chao 1 index, (c) the Shannon index, (d) PCoA analyses based on the weighted UniFrac distance, and (e) NMDS analyses based on the BrayCurtis distance. Data are expressed as the mean ± SD (*n* = 4 rats/group). ^*∗∗*^*p* < 0.01 vs. NC group and ^###^*p* < 0.001 vs. DC group.

16S rRNA gene amplicon sequencing was applied to detect the faecal bacterial community of T2DM rats. A total of 824,026 valid sequences were obtained, and 3694 operational taxonomic units (OTUs) were delineated for further analysis. A dilution curve was used to compare the species richness of samples with different sequencing data. As shown in Figure 8(a), most of the sample curves tended to be flat, indicating that the number of OTUs in the sample tended to be stable, and the sequencing data amount could reflect the total number of OTUs in the sample.

The Chao1 and Shannon *a*-diversity indexes were the main indexes reflecting the richness and diversity of intestinal bacterial species, respectively. The data showed that the Chao1 index in the DC group was significantly higher than that in the NC group (Figure 8(b)), but the Shannon index was significantly reduced (Figure 8(c)). After treatment with SC and metformin, both indicators declined markedly, which indicated that the intervention drugs could inhibit the richness and diversity of the gut microbiota.

Principal coordinate analysis (PCoA) and nonmetric multidimensional scaling (NMDS) are the main indicators of *ß*-diversity of the gut microbiota, and the distance between points reflected the difference among samples. As shown in Figures 8(d) and 8(e), the structural characteristics of the bacterial community in the high, medium, and low SC groups were similar, but there was no tendency to recover to the levels of the NC group, which suggested that SC could reshape the gut microbiota rather than restore its structural characteristics.

### 3.7. Analysis of the Bacterial Community Composition

**Figure 9:**
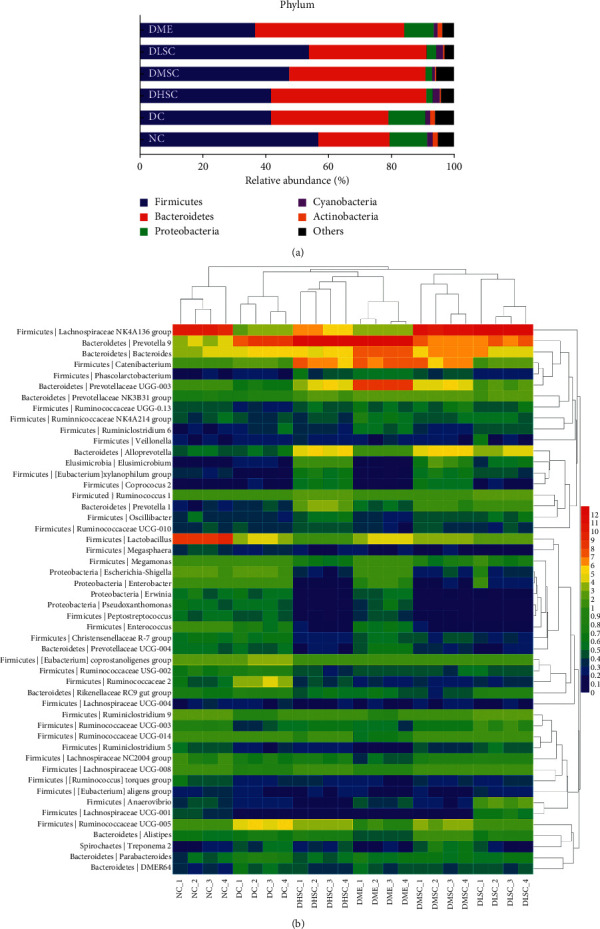
Effect of SC on the community composition of the gut microbiota. Community composition changes in the gut microbiota at (a) the phylum level and (b) the genus level. Data are expressed as the mean (*n* = 4 rats/group).

Compared to the NC group, the intestinal flora abundance in the DC group changed obviously. For example, the abundant levels of Proteobacteria, Enterobacteriaceae, *Enterococcus*, *Escherichia-Shigella*, and *Enterobacter* increased, while the Firmicutes/Bacteroidetes ratio and the levels of some gut flora, such as the *Lachnospiraceae NK4A136* group and *Lactobacillus*, showed downward trends. The regulatory effect of SC on the abundance of different intestinal bacteria was significant. Briefly, at the phylum level (Figure 9(a)), SC could reduce the abundance of Proteobacteria. At the family level (Table 1), it could increase the abundance of Lachnospiraceae, Prevotellacae, and Erysipelototrichaeae, and with an increase in the herbal dosage, the effect of SC on Prevotellacae proliferation was gradually enhanced, while the effect of promoting Lachnospiraceae proliferation was gradually reduced. SC could also inhibit the abundance of Enterobactaceae, Lactobacillaceae, and Biofidobacteria. At the genus level (Figure 9(b) and Table 1), the abundance of *Lachnospiraceae NK4A136* group*, Prevotella 9, Prevotellacae UCG-003, Alloprevotella*, *Prevotella NK3B31* group, and *Prevotella 1* increased, and the abundance of *Lactobacillus*, *Escherichia-Shigella*, *Enterobacter*, and *Enterococcus* decreased. In addition, the positive control drug metformin had a significant regulatory effect on the gut microbiota; for example, it could increase the abundance of Prevotellaceae, Lachnospiraceae, *Prevotella 9*, *Prevotellaceae UCG-003*, and *Prevotellaceae NK3B31* group.

## 4. Discussion

**Figure 10:**
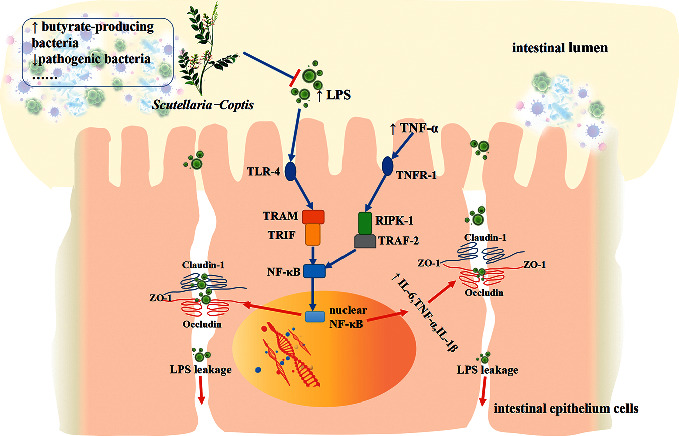
SC alleviates intestinal mucosal barrier damage in diabetic rats by inhibiting inflammation and modulating the gut microbiota. Briefly, SC could inhibit the proliferation of the LPS-producing bacteria, promote the increase in the butyrate-producing bacteria, and restrain the inflammatory response through the TLR-4/TRIF and TNFR-1/NF-*κ*B signalling pathways, thus playing a role in intestinal mucosal protection.

This study explored the molecular mechanism of the herbal medicine SC in ameliorating T2DM from the perspective of the gut microbiota and the epithelial mucosa. The experimental results showed that SC could improve the damage to the intestinal mucosa caused by a high-fat diet and STZ, inhibit the leakage of LPS and regulate the expression of the intestinal tight junction proteins ZO-1, occluding, and claudin-1. Further studies suggested that the mechanism of SC in protecting the intestinal mucosa barrier might be related to the inhibition of inflammatory responses involving the TLR-4/TRIF and TNFR-1/NF-*κ*B signalling pathways. The results of 16S rRNA high-throughput sequencing showed that SC could markedly reduce the abundance of the intestinal conditioned pathogens Proteobacteria, *Enterobacteriaceae, Enterococcus, Escherichia-Shigella,* and *Enterobacter* and promote the proliferation of some genera belonging to Lachnospiraceae and Prevotellacae (Figure 10).

With the deepening of scientific studies, researchers have come to realize that the gut is not only a simple digestive and absorption organ but also an important contributor to the maintenance of homeostasis of human metabolism [39]. LPS leakage from the damaged intestinal mucosa is an important factor leading to chronic systemic inflammation and glucose metabolism disorders [9]. LPS can lead to the production of IL-1*β* in islet cells, activate a variety of inflammatory factors, and ultimately induce cell apoptosis [40]; moreover, LPS-mediated inflammation can also destroy the insulin signal transduction pathway and reduce insulin utilization efficiency [10]. In this study, the improvement in insulin utilization efficiency and insulin secretion by SC may be closely related to the inhibition of serum LPS and inflammatory factor levels.

Serum markers, histological staining, and immunohistochemistry analysis were used to evaluate the repairing effect of SC on intestinal barrier damage. Dao is a highly active enzyme in mammalian intestinal villus cells [41], and D-LA is the fermentation product of intestinal bacteria [42]. Similar to LPS, the increase in these two indicators also indicates higher intestinal permeability. The tight junction is the main connection mode between intestinal epithelial cells and plays a crucial role in maintaining the normal structure and function of the intestinal barrier [38]. Claudins, an important component of transmembrane proteins, are the main skeleton proteins of the tight junction complex, and claudin-1, part of the claudin family, has a strong sealing effect on the gap between cells. In addition, another transmembrane protein, occludin, and the peripheral membrane protein ZO-1 can interact directly with claudins and actin and are crucial to tight junction assembly and maintenance [38]. In the present study, SC had an obvious upregulation effect on the abovementioned proteins, which suggested that the tight junction may be the vital target of SC's intestinal protective effect.

The intestinal inflammatory response is a crucial factor in destroying intestinal epithelial cells and tight junctions [43]. TLR-4, the receptor of LPS, can mediate the development of innate immunity, and its activation can promote NF-*κ*B nuclear translocation through MyD88 or TRIF signalling pathways [23]. Recently, Zhang and his colleagues [44] confirmed that SC could inhibit the expression of TLR-4 and MyD88 in the colons of diabetic mice, but until now, there has been no related study on the effect of SC on the TRIF pathway. With the participation of TRAM, TLR-4 recruits TRIF and further activates tumour necrosis factor receptor-related factor 6 (TRAF-6) as well as transforming growth factor-*β*-activated kinase-1 (TAK-1), catalysing the phosphorylation of inhibitor of kappa B (I*κ*B) protein and promoting the translocation of NF-*κ*B to the nucleus. In addition, the release of TNF-*α* in large amounts can further activate NF-*κ*B in a receptor-dependent manner. Specifically, the combination of TNF-*α* and its receptor TNFR-1 can promote the formation of a membrane-binding complex containing TNF receptor-associated proteins with a death domain (TRADD), TRAF, and RIPK-1 and can then mediate cell apoptosis as well as the inflammatory response involving NF-*κ*B. In this study, the expression levels of the above proteins were upregulated in the DC group, and SC played an inhibitory role, which suggested that SC could inhibit intestinal inflammation through the TLR-4/TRIF and TNFR-1/NF-*κ*B pathways.

The interaction between the intestinal flora and the intestinal mucosa is a key factor in maintaining intestinal barrier homeostasis [11]. In this study, 16S rRNA high-throughput sequencing was used to detect the gut microbiota, and as expected, the regulation of SC on the gut microbiota might be one of the significant reasons for its anti-inflammatory effect. First, SC had a strong inhibitory effect on potential enteropathogenic bacteria, such as Proteobacteria, Enterobacteriaceae, *Enterobacter*, *Escherichia-Shigella*, and *Enterococcus*. Proteobacteria is one of the most abundant phyla and is regarded as a potential diagnostic microbial signature of epithelial dysfunction [45]. Studies confirmed that there was a mutually reinforcing relationship between the increase in its abundance and the intestinal inflammatory response [44, 45]. Enterobacteriaceae belonging to Proteobacteria contain many gram-negative pathogenic bacterial genera, and in this study, the increases in *Escherichia-Shigella* and *Enterobacter* may be closely related to intestinal epithelial damage and metabolic disorders. Previous research found that a long-term Western diet led to the enrichment of adherent-invasive *Escherichia-Shigella* as well as increases in intestinal inflammation and permeability [20]. Moreover, the virulence factor *Escherichia-Shigella* can also directly destroy intestinal tight junction proteins and actin cytoskeleton and then damage the intestinal barrier [46]. *Enterobacter*, another major producer of LPS, may also promote chronic inflammation in the intestine and the whole body. Fei and Zhao [47] found that there was a correlation between the increase in *Enterobacter*'s abundance and metabolic parameter abnormalities, and functional food prescription could regulate glycolipid metabolism by inhibiting the proliferation of this bacterium. In addition, the increase in *Enterococcus* in diabetes rodent models/patients was also found in our and others' studies [21], and as a potential pathogenic bacteria, the enrichment of *Enterococcus* will also compromise the epithelial barrier and contribute to intestinal inflammation. As described above, SC strongly inhibited the proliferation of these potential proinflammatory bacteria, thus hindering the activation of immune inflammation.

Promoting the proliferation of butyrate-producing bacteria, such as Lachnospiraceae and Prevotellaceae, is another important aspect of SC in regulating the gut microbiota. Lachnospiraceae contains a variety of butyrate-producing bacteria, and in this study, the *Lachnospiraceae NK4A136* group and *Coprococcus 2* showed the most obvious upward trends in the SC treatment groups. In addition, it is possible that bacteria in Prevotellaceae can produce butyrate via succinate [48], and in particular, the genera *Prevotella 9*, *Alloprevotella*, *Prevotellaceae UCG-003*, and *Prevotellaceae NK3B31* increased significantly in the SC intervention groups. Previous studies also showed that SC or its active ingredients could promote the proliferation of microorganisms in Prevotellaceae. Xiao et al.'s [49] study found that, after treating diabetic rats with SC for 30 days, the abundance of intestinal *Prevotellaceae UCG-001*, rather than the above genera we found, was significantly increased; furthermore, Xiexin decoction, composed of SC and *Rheum officinale*, could effectively promote the proliferation of the *Prevotellaceae NK3B31* group and *Alloprevotella* [50]. Meanwhile, metformin, the first-line drug for treating T2DM, has also been confirmed to have a clear effect in regulating the gut microbiota, and in the present study, the abundance of the Prevotellaceae family and *Prevotella 9, Prevotellaceae UCG-003*, and *Prevotellaceae NK3B31* group genera also increased markedly in the DME treatment group, which suggested that there were similar pharmacological effects between SC and metformin in promoting the proliferation of microorganisms in Prevotellaceae.

However, the potential side effects of SC on the gut microbiota cannot be ignored. It has been found that *Scutellaria* and *Coptis* both have remarkable antibacterial effects [51, 52], and in this study, the total amount and diversity of the gut microbiota were significantly inhibited in the SC intervention groups; at the same time, the abundance levels of some beneficial genera, such as *Lactobacillus*, were also markedly reduced. Therefore, the effect of SC on the gut microbiota was multidimensional, as it could not only inhibit harmful bacteria but also destroy the normal structure of the intestinal flora. In future research, we intend to add some probiotics or prebiotics to the basic formula of SC to eliminate the adverse effect of SC on the gut microbiota.

## 5. Conclusion

Taken together, the protective effect of SC on the intestinal epithelial mucosa may be related to the inhibition of inflammation, involving the TLR-4/TRIF and TNFR-1/NF-*κ*B signalling pathways, and to the regulation of the gut microbiota. By repairing the damaged intestinal barrier, SC can reduce the amounts of LPS and inflammatory factors in the circulation, thus achieving a therapeutic effect in T2D.

## Figures and Tables

**Table 1:** Differentially abundant features analysis at the family or genus level.

Family/genus	NC (%) mean ± SD	DC (%) mean ± SD	DHSC (%) mean ± SD	DMSC (%) mean ± SD	DLSC (%) mean ± SD	DME (%) mean ± SD
*Family*						
Enterobacteriaceae	2.43 ± 0.32	4.21 ± 0.33^*∗∗∗*^	0.52 ± 0.07^###^	0.55 ± 0.15^###^	1.04 ± 0.78^###^	3.34 ± 0.3^##^
Lachnospiraceae	21 ± 0.54	8.56 ± 0.42^*∗∗∗*^	13.72 ± 0.99^###^	18.82 ± 0.21^###^	24.58 ± 0.99^###^	7.81 ± 0.28
Prevotellacae	7.37 ± 0.39	12.06 ± 0.61^*∗∗∗*^	28.66 ± 1.22^###^	21.03 ± 0.51^###^	16.71 ± 1.04^###^	28.33 ± 1.09^###^
Erysipelototrichaeae	1.73 ± 0.13	2.43 ± 0.57	6.56 ± 0.97^###^	6.49 ± 0.51^###^	2.58 ± 0.35	7.5 ± 0.36^###^
Lactobacillaceae	8.82 ± 0.45	3.98 ± 0.07^*∗∗∗*^	1.84 ± 0.14^###^	3.31 ± 0.12^###^	2.24 ± 0.04^###^	4.12 ± 0.23
Biofidobacteria	0.57 ± 0.09	0.61 ± 0.08	0.13 ± 0.03^###^	0.13 ± 0.02^###^	0.26 ± 0.3^##^	0.5 ± 0.06

*Genus*						
*Enterococcus*	0.44 ± 0.12	0.73 ± 0.1^*∗∗∗*^	0.15 ± 0.05^###^	0.15 ± 0.06^###^	0.15 ± 0.02^###^	0.6 ± 0.04^#^
*Escherichia-Shigella*	1.17 ± 0.15	2.08 ± 0.14^*∗∗∗*^	0.26 ± 0.05^###^	0.28 ± 0.1^###^	0.44 ± 0.32^###^	1.54 ± 0.14^###^
*Enterobacter*	0.44 ± 0.17	1.32 ± 0.14^*∗∗∗*^	0.15 ± 0.01^###^	0.18 ± 0.04^###^	0.45 ± 0.42^###^	1.04 ± 0.13
*LachnospiraceaeNK4A136* group	10.39 ± 0.49	3.18 ± 0.25^*∗∗∗*^	5.6 ± 0.54^###^	11.25 ± 0.19^###^	15.01 ± 0.78^###^	3.48 ± 0.04
*Lactobacillus*	8.74 ± 0.46	3.93 ± 0.08^*∗∗∗*^	1.83 ± 0.14^###^	3.3 ± 0.11^##^	2.23 ± 0.42^###^	4.06 ± 0.23
*Prevotella 1*	0.27 ± 0.09	0.37 ± 0.04	2.96 ± 0.32^###^	1.03 ± 0.05^###^	1.13 ± 0.06^###^	0.5 ± 0.11
*Prevotella 9*	3.92 ± 0.23	8.48 ± 0.44^*∗∗∗*^	13.42 ± 0.67^###^	6.51 ± 0.55^###^	7.06 ± 0.54^###^	14.06 ± 0.66^###^
*Prevotellacae UCG-003*	1.15 ± 0.06	0.65 ± 0.03^*∗*^	4.53 ± 0.56^###^	4.72 ± 0.3^###^	1.94 ± 0.22^###^	8.39 ± 0.32^###^
*Alloprevotella*	0.47 ± 0.07	0.53 ± 0.07	4.83 ± 0.39^###^	5.25 ± 0.46^###^	3.9 ± 0.25^###^	1.71 ± 0.08^###^
*Prevotellacae NK3B31 group*	0.74 ± 0.06	0.89 ± 0.08	2.05 ± 0.2^###^	2.58 ± 0.09^###^	2.15 ± 0.33^###^	2.24 ± 0.11^###^

*Note.* Data are expressed as the mean ± SD (*n* = 4 rats/group). ^*∗*^*p* < 0.05, ^*∗∗*^*p* < 0.01, ^*∗∗∗*^*p* < 0.001 vs. NC group, ^#^*p* < 0.05, ^##^*p* < 0.01, and ^###^*p* < 0.001 vs. DC group.

## Data Availability

The data used to support the results of this study are available from the corresponding author upon request.

## Supplementary Materials

Supplementary Materials Table S1: the formula composition of the high-fat diet; Figure S1: fingerprints of the *Scutellaria baicalensis* and the *Coptis chinensis*.

## References

[1] International Diabetes Federation (2019). *IDF Diabetes Atlas*.

[2] Wellen K. E., Hotamisligil G. S. (2005). Inflammation, stress, and diabetes. *Journal of Clinical Investigation*.

[3] Donath M. Y. (2013). Targeting inflammation in the treatment of type 2 diabetes. *Diabetes, Obesity and Metabolism*.

[4] Cavelti-Weder C., Babians-Brunner A., Keller C. (2012). Effects of gevokizumab on glycemia and inflammatory markers in type 2 diabetes. *Diabetes Care*.

[5] Pradhan A. D., Manson J. E., Rifai N., Buring J. E., Ridker P. M. (2001). C-reactive protein, interleukin 6, and risk of developing type 2 diabetes mellitus. *JAMA*.

[6] Laaksonen D. E., Niskanen L., Nyyssönen K. (2004). C-reactive protein and the development of the metabolic syndrome and diabetes in middle-aged men. *Diabetologia*.

[7] Odegaard J. I., Chawla A. (2013). Pleiotropic actions of insulin resistance and inflammation in metabolic homeostasis. *Science*.

[8] Eguchi K., Nagai R. (2017). Islet inflammation in type 2 diabetes and physiology. *Journal of Clinical Investigation*.

[9] Cani P. D., Osto M., Geurts L., Everard A. (2012). Involvement of gut microbiota in the development of low-grade inflammation and type 2 diabetes associated with obesity. *Gut Microbes*.

[10] Cani P. D., Bibiloni R., Knauf C. (2008). Changes in gut microbiota control metabolic endotoxemia-induced inflammation in high-fat diet-induced obesity and diabetes in mice. *Diabetes*.

[11] Vancamelbeke M., Vermeire S. (2017). The intestinal barrier: a fundamental role in health and disease. *Expert Review of Gastroenterology & Hepatology*.

[12] Horton F., Wright J., Smith L., Hinton P. J., Robertson M. D. (2014). Increased intestinal permeability to oral chromium (51Cr)-EDTA in human type 2 diabetes. *Diabetic Medicine*.

[13] Zhong H. J., Yuan Y., Xie W. R., Chen M. H., He X. X. (2016). Type 2 diabetes mellitus is associated with more serious small intestinal mucosal injuries. *PLoS One*.

[14] Shen L., Ao L., Xu H. (2019). Poor short-term glycemic control in patients with type 2 diabetes impairs the intestinal mucosal barrier: a prospective, single-center, observational study. *BMC Endocrine Disorders*.

[15] Cox A. J., Zhang P., Bowden D. W. (2017). Increased intestinal permeability as a risk factor for type 2 diabetes. *Diabetes & Metabolism*.

[16] Bron P. A., Kleerebezem M., Brummer R.-J. (2017). Can probiotics modulate human disease by impacting intestinal barrier function?. *British Journal of Nutrition*.

[17] Li Y., Lv L., Ye J. (2019). Bifidobacterium adolescentis CGMCC 15058 alleviates liver injury, enhances the intestinal barrier and modifies the gut microbiota in D-galactosamine-treated rats. *Applied Microbiology and Biotechnology*.

[18] Ottman N., Reunanen J., Meijerink M. (2017). Pili-like proteins of *Akkermansia muciniphila* modulate host immune responses and gut barrier function. *PLoS One*.

[19] Bach Knudsen K., Lærke H., Hedemann M. (2018). Impact of diet-modulated butyrate production on intestinal barrier function and inflammation. *Nutrients*.

[20] Martinez-Medina M., Denizot J., Dreux N. (2014). Western diet induces dysbiosis with increasedE coliin CEABAC10 mice,alters host barrier function favouring AIEC colonisation. *Gut*.

[21] Qin J., Li Y., Cai Z. (2012). A metagenome-wide association study of gut microbiota in type 2 diabetes. *Nature*.

[22] Lin L., Zhang J. (2017). Role of intestinal microbiota and metabolites on gut homeostasis and human diseases. *BMC Immunology*.

[23] Fresno M., Alvarez R., Cuesta N. (2011). Toll-like receptors, inflammation, metabolism and obesity. *Archives of Physiology and Biochemistry*.

[24] Wong J., Garcia-Carbonell R., Zelic M. (2020). RIPK1 mediates TNF-induced intestinal crypt apoptosis during chronic NF-*κ*B activation. *Cellular and Molecular Gastroenterology and Hepatology*.

[25] Watson A. J. M., Hughes K. R. (2012). TNF-*α*-induced intestinal epithelial cell shedding: implications for intestinal barrier function. *Annals of the New York Academy of Sciences*.

[26] Shah M. A., Jakkawanpitak C., Sermwittayawong D., Panichayupakaranant P. (2018). Rhinacanthins-rich extract enhances glucose uptake and inhibits adipogenesis in 3T3-L1 adipocytes and L6 myotubes. *Pharmacognosy Magazine*.

[27] Shah M. A., Reanmongkol W., Radenahmad N., Khalil R., Ul-Haq Z., Panichayupakaranant P. (2019). Anti-hyperglycemic and anti-hyperlipidemic effects of rhinacanthins-rich extract from Rhinacanthus nasutus leaves in nicotinamide-streptozotocin induced diabetic rats. *Biomedicine & Pharmacotherapy*.

[28] Shah M. A., Khalil R., Ul-Haq Z. (2017). *α*-Glucosidase inhibitory effect of rhinacanthins-rich extract from Rhinacanthus nasutus leaf and synergistic effect in combination with acarbose. *Journal of Functional Foods*.

[29] Ryuk J. A., Lixia M., Cao S., Ko B.-S., Park S. (2017). Efficacy and safety of Gegen Qinlian decoction for normalizing hyperglycemia in diabetic patients: a systematic review and meta-analysis of randomized clinical trials. *Complementary Therapies in Medicine*.

[30] Chandirasegaran G., Elanchezhiyan C., Ghosh K., Sethupathy S. (2017). Berberine chloride ameliorates oxidative stress, inflammation and apoptosis in the pancreas of Streptozotocin induced diabetic rats. *Biomedicine & Pharmacotherapy*.

[31] You W., Wang K., Yu C., Song L. (2018). Baicalin prevents tumor necrosis factor-*α*−induced apoptosis and dysfunction of pancreatic *β*-cell line Min6 via upregulation of miR-205. *Journal of Cellular Biochemistry*.

[32] Zhang C.-H., Sheng J.-Q., Sarsaiya S. (2019). The anti-diabetic activities, Gut microbiota composition, the anti-inflammatory effects of scutellaria-coptis herb couple against insulin resistance-model of diabetes involving the toll-like receptor 4 signaling pathway. *Journal of Ethnopharmacology*.

[33] Gong J., Hu M., Huang Z. (2017). Berberine attenuates intestinal mucosal barrier dysfunction in Type 2 diabetic rats. *Frontiers in Pharmacology*.

[34] Cui L., Feng L., Zhang Z. H., Jia X. B. (2014). The anti-inflammation effect of baicalin on experimental colitis through inhibiting TLR4/NF-*κ*B pathway activation. *International Immunopharmacology*.

[35] Xu M., Yue R. S., Yang M. Y., Yang X., Wu T. C., Li J. N. (2018). Effects of banxia xiexin decoction on intestinal flora and inflammatory factors of diabetic gastroparesis rats. *Chinese Traditional and Herbal Drugs*.

[36] Srinivasan K., Viswanad B., Asrat L., Kaul C. L., Ramarao P. (2005). Combination of high-fat diet-fed and low-dose streptozotocin-treated rat: a model for type 2 diabetes and pharmacological screening. *Pharmacological Research*.

[37] Wallace T. M., Levy J. C., Matthews D. R. (2004). Use and abuse of HOMA modeling. *Diabetes Care*.

[38] Turner J. R. (2009). Intestinal mucosal barrier function in health and disease. *Nature Reviews Immunology*.

[39] de Kort S., Keszthelyi D., Masclee A. A. M. (2011). Leaky gut and diabetes mellitus: what is the link?. *Obesity Reviews*.

[40] Liu H., Yin J.-J., Cao M.-M., Liu G.-D., Su Y., Li Y.-B. (2017). Endoplasmic reticulum stress induced by lipopolysaccharide is involved in the association between inflammation and autophagy in INS-1 cells. *Molecular Medicine Reports*.

[41] Wolvekamp M. C. J., de Bruin R. W. F. (1994). Diamine oxidase: an overview of historical, biochemical and functional aspects. *Digestive Diseases*.

[42] Bischoff S. C., Barbara G., Buurman W. (2014). Intestinal permeability--a new target for disease prevention and therapy. *BMC Gastroenterology*.

[43] Sánchez de Medina F., Romero-Calvo I., Mascaraque C., Martínez-Augustin O. (2014). Intestinal inflammation and mucosal barrier function. *Inflammatory Bowel Diseases*.

[44] Litvak Y., Byndloss M. X., Tsolis R. M., Bäumler A. J. (2017). Dysbiotic proteobacteria expansion: a microbial signature of epithelial dysfunction. *Current Opinion in Microbiology*.

[45] Rizzatti G., Lopetuso L. R., Gibiino G., Binda C., Gasbarrini A. (2017). Proteobacteria: a common factor in human diseases. *BioMed Research International*.

[46] Pawłowska B., Sobieszczańska B. M. (2017). Intestinal epithelial barrier: the target for pathogenic *Escherichia coli*. *Advances in Clinical and Experimental Medicine: Official Organ Wroclaw Medical University*.

[47] Fei N., Zhao L. (2013). An opportunistic pathogen isolated from the gut of an obese human causes obesity in germfree mice. *The ISME Journal*.

[48] Esquivel-Elizondo S., Ilhan Z. E., Garcia-Peña E. I., Krajmalnik-Brown R. (2017). Insights into butyrate production in a controlled fermentation system via gene predictions. *mSystems*.

[49] Xiao S., Liu C., Chen M. (2020). Scutellariae radix and coptidis rhizoma ameliorate glycolipid metabolism of type 2 diabetic rats by modulating Gut microbiota and its metabolites. *Applied Microbiology and Biotechnology*.

[50] Wei X., Tao J., Xiao S. (2018). Xiexin Tang improves the symptom of type 2 diabetic rats by modulation of the Gut microbiota. *Scientific Reports*.

[51] Mekseepralard C., Poonpon P., Kamkaen N. (2007). Antibacterial activities of *Coptis chinensis* and *Glycyrrhiza glabra* against seven bacteria associated with human gastrointestinal infections. *Planta Medica*.

[52] Lu Y., Joerger R., Wu C. (2011). Study of the chemical composition and antimicrobial activities of ethanolic extracts from roots of Scutellaria baicalensis Georgi. *Journal of Agricultural and Food Chemistry*.

