# Supplementary material for: The Herbal Medicine Scutellaria-Coptis Alleviates Intestinal Mucosal Barrier Damage in Diabetic Rats by Inhibiting Inflammation and Modulating the Gut Microbiota
Source: Evid Based Complement Alternat Med. 2020 Nov 8;2020:4568629. doi: 10.1155/2020/4568629 (PMC7669352; doi:10.1155/2020/4568629)

| The formula composition of the high fat diet | |
| --- | --- |
| Basic rat diet | 74% |
| lard | 10% |
| sucrose | 7% |
| casein | 5% |
| fish meal | 2% |
| Maltodextrin | 2% |
| methionine | 0.1% |

**Table S1: The formula composition of the high fat diet**

**Figure S1：Fingerprints of the *Scutellaria baicalensis* and the *Coptis chinensis***


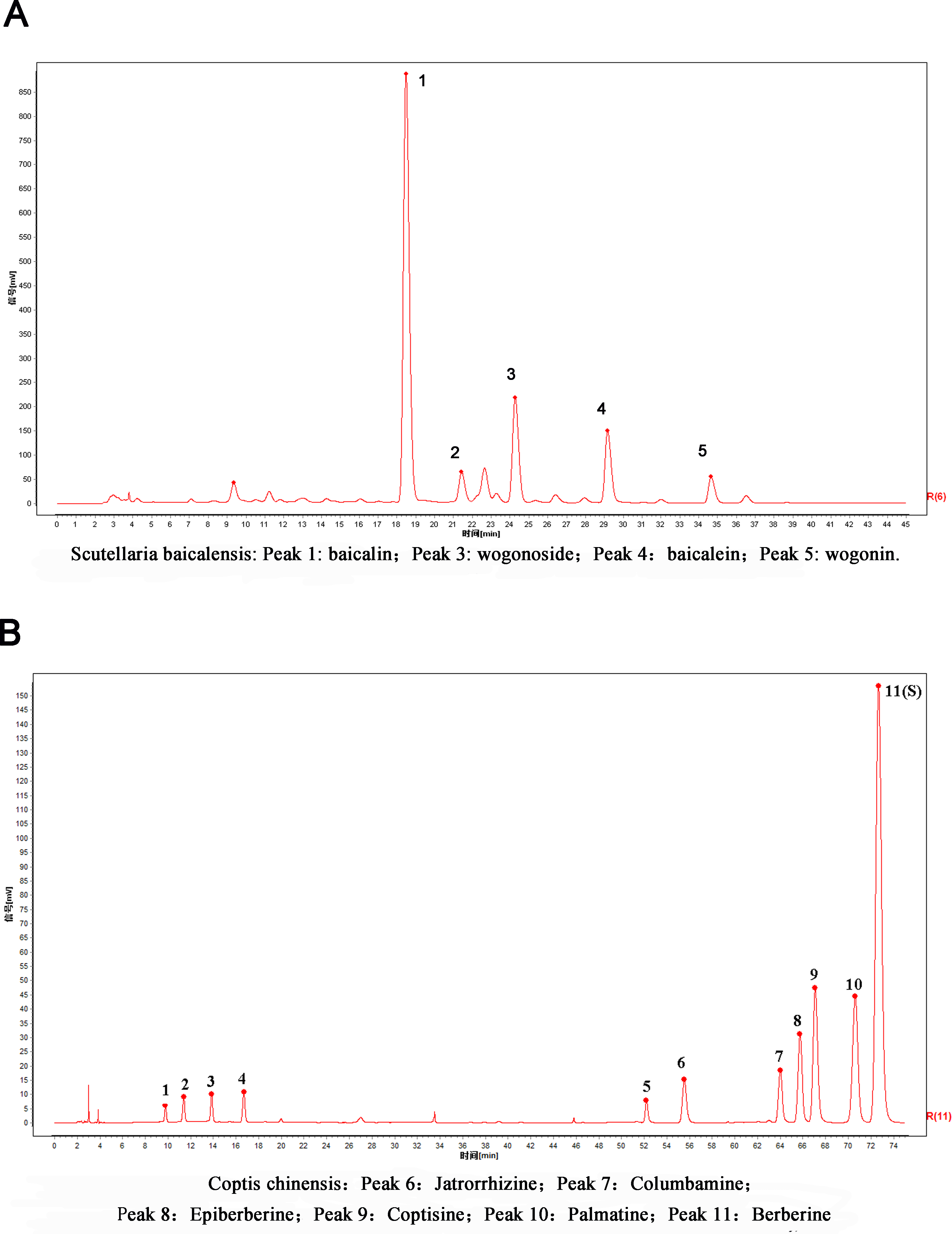

Supplement: Supplementary Materials — Table S1: the formula composition of the high-fat diet; Figure S1: fingerprints of the Scutellaria baicalensis and the Coptis chinensis. [file 4568629.f1.doc]
